# Age modulates, religious coping mediates: the role of attitude to self in cancer patients’ quality of life

**DOI:** 10.3389/fpsyg.2025.1558236

**Published:** 2025-02-12

**Authors:** Maria-Chidi C. Onyedibe, Runcie C.W. Chidebe, Barbara L. Andersen, Uzoamaka F. Ugwoke, Lawrence Ejike Ugwu

**Affiliations:** ^1^Department of Psychology, University of Nigeria, Nsukka, Nigeria; ^2^Project PINK BLUE-Health and Psychological Trust Centre, Abuja, Nigeria; ^3^Department of Sociology and Gerontology, Miami University, Oxford, OH, United States; ^4^Scripps Gerontology Center, Miami University, Oxford, OH, United States; ^5^Department of Psychology, Ohio State University, Lima, OH, United States; ^6^Psychology Department, Enugu State University of Science and Technology, Enugu, Nigeria; ^7^Renaissance University, Ugbawka, Nigeria; ^8^Faculty of Humanities, North-West University, Mafikeng, South Africa

**Keywords:** age, attitude towards self, cancer, health-related quality of life, religious coping

## Abstract

**Background:**

Cancer significantly impacts psychological well-being and health-related quality of life (HRQoL). This is particularly evident in low and middle-income countries (LMICs) where healthcare disparities exacerbate distress. Cognitive vulnerabilities, including negative generalization, self-criticism, and high standards, may influence HRQoL through their effects on coping mechanisms. Religious coping, shaped by cultural norms in LMICs, may mediate these relationships, with age as a potential moderator.

**Objective:**

This study examines how attitudes toward self (ATS), religious coping, and age influence HRQoL among cancer patients in an LMIC context.

**Methods:**

A cross-sectional study of 565 cancer patients in a University Teaching Hospital, Southwest Nigeria completed a standardized and validated self-report measures of ATS, HRQoL, and religious coping. Mediation and moderation analyses were conducted using SPSS (Version 29) and SmartPLS (Version 4).

**Results:**

Negative generalization (β = −0.25, *p* < 0.001) and self-criticism (β = −0.19, *p* < 0.001) reduced physical well-being, while high standards positively influenced emotional well-being (β = 0.27, *p* < 0.001) but negatively impacted functional well-being (β = −0.13, *p* < 0.01). Negative religious coping (NRC) mediated the relationships between ATS and HRQoL, amplifying negative effects on physical (β = −0.25, *p* < 0.001) and functional well-being (β = −0.32, *p* < 0.001). Age moderated these relationships, with older patients showing sharper declines in physical (β = −0.09, *p* < 0.01) and functional well-being (β = −0.10, *p* < 0.01). Positive religious coping had limited effects.

**Conclusion:**

The study highlights the significant impact of cognitive vulnerabilities and maladaptive religious coping on the HRQoL of older cancer patients in LMICs. These findings underscore the urgent need for tailored interventions that integrate cognitive-behavioral therapy (CBT) with culturally and religiously sensitive approaches to improve patient outcomes. Policymakers and healthcare providers should prioritize training and resource allocation to address these challenges. Future research should focus on longitudinal patterns of coping, as well as gender-related differences, to develop more inclusive and effective strategies for enhancing the well-being of cancer patients.

## Introduction

Cancer is a profoundly distressing disease that significantly impacts psychological well-being and health-related quality of life (HRQoL). Research consistently shows that cancer patients experience heightened psychological distress, including depression, which further compromises HRQoL (Andreu et al., 2024; Taylor et al., 2024). This is especially problematic for patients in low- and middle-income countries (LMICs), where healthcare disparities exacerbate cancer challenges (Naamala et al., 2024). Cancer survivorship rates are generally lower in low- and middle-income countries compared to high-income countries, largely due to disparities related to national development and healthcare infrastructure (Soerjomataram et al., 2023). In Nigeria, specifically, lower cancer survival rates have been attributed to several factors, including late cancer detection (Jedy-Agba et al., 2016), limited access to cancer screening (Sharma et al., 2021), and inadequate availability of comprehensive treatment options (Olasehinde et al., 2021). For example, the lack of advanced radiotherapy treatment machines and other essential cancer treatment facilities (Knapp et al., 2019; Aruah et al., 2023) severely limits access to effective care. These systemic healthcare challenges in Nigeria contribute to increased psychological distress and a lower health-related quality of life among cancer patients. Among these psychological distress, depression has been identified as a critical factor negatively influencing HRQoL (Alwhaibi et al., 2023). Cognitive theories of depression provide a framework for understanding how individuals interpret and respond to stressful life events, such as a cancer diagnosis. Beck’s cognitive triad (Beck, 1967; Beck et al., 1987) posits that individuals with depression often have distorted and negative beliefs about themselves, their environment, and the future. These cognitions, collectively called cognitive vulnerabilities, are stable tendencies to interpret information in a biased manner, especially during stress (Zhong et al., 2011). Cognitive vulnerabilities, including negative generalization, self-criticism, and high personal standards, have been consistently linked to the development of depressive symptoms (Abramson et al., 2002).

Attitudes toward the self, encompassing these cognitive vulnerabilities, have been extensively studied in non-clinical populations (Carver, 1998; Eisner et al., 2008), but little is known about their role in cancer patients. This gap is significant, considering the high prevalence of depression (Alwhaibi et al., 2023) and its association with HRQoL. Furthermore, the cultural context of LMICs, where communal values (expressed through shared social life, commitment to the common good, and mutual obligations etc., Awoniyi, 2015) and spirituality often influence coping mechanisms (Ozcan et al., 2021) remains underexplored among cancer patients. Addressing this gap is crucial to designing culturally relevant interventions for improving HRQoL among cancer patients.

Religious coping, a commonly utilized strategy in low- and middle-income countries (LMICs), may serve as a significant coping strategy for enhancing the HRQoL of cancer patients. Religious coping refers to using religious behaviors and beliefs to manage stress and adversity (Pargament et al., 2011). It can take two forms: positive religious coping (PRC), characterized by a secure attachment to the sacred and a benevolent worldview, and negative religious coping, referring to spiritual struggles, tension, and a punitive view of the divine. Research among cancer patients has shown that religion and spirituality are major coping strategies (Arbinaga et al., 2021) being used by cancer patients. For example, a recent qualitative study, (de Medeiros et al., 2024) found that religious coping is the most commonly employed strategy among cancer patients with strong faith in God. Moreover, the relationship between positive and negative religious coping and HRQoL have been identified. For instance, studies among cancer patients show that positive religious coping is often associated with better HRQoL, while negative religious coping predicts poorer HRQoL (Bruce et al., 2020; Sarwoko et al., 2022). However, findings remain mixed, with some studies suggesting that negative religious coping may have a more significant role than positive religious coping in influencing HRQoL (Maier et al., 2022; Pagán-Torres et al., 2021).

These mixed findings underscore the need for further research, particularly in low- and middle-income countries where studies remain scarce. In addition, we propose that religious coping could serve as a mediator variable. Although previous research has emphasized the importance of including religious coping as a mediator or moderator (Park et al., 2017), few studies have explored this area. Among non-cancer populations, Maier et al. (2022) found that negative religious coping, but not positive religious coping, mediated the relationship between spiritual needs and well-being among German migrants. Similarly, Pagán-Torres et al. (2021) reported that negative religious coping mediated the link between perceived stress and psychological well-being in Puerto Rican adults, whereas positive religious coping did not. In cancer populations, only one relevant study was identified. For instance, Okwuosa et al. (2024) demonstrated that negative religious coping mediated the relationship between perceived stress and HRQoL in cancer patients, whereas positive religious coping did not. Further studies are needed to explore the mediating role of positive and negative religious coping in these relationships, emphasizing the rationale for the present study.

In studying religious coping, it is relevant to consider the individual’s age, as it is a critical factor influencing HRQoL. Older individuals may employ different forms of religious coping than younger individuals, which could impact their health-related quality of life (HRQoL). Age has been associated with various domains of HRQoL, with younger cancer patients often reporting worse physical and emotional functioning compared to older patients, who may experience improvements in areas like social functioning (Quinten et al., 2015). Andreu et al. (2024) found that younger cancer patients (18–45 years) reported worse overall quality of life (QoL), including more negative feelings, cognitive and social issues, and distress, while some aspects of QoL appeared to improve with age. Age has been identified as a moderator between psychological constructs and HRQoL in both non-clinical (Hao et al., 2024; Benton and Hutchins, 2024) and clinical samples (Kłosowska et al., 2023; Skwirczyńska et al., 2023). However, its moderating role among cancer patients remains underexplored, highlighting the need for this study. We identified only one meta-analysis on Randomized Clinical Trials (RCTs) involving cancer patients (Kalter et al., 2018). In this individual patient data meta-analysis of 22 RCTs, Kalter et al. (2018) reported that the effects of psychosocial interventions on QoL, particularly emotional and social functioning, were greater for younger patients compared to older patients. Despite this, the moderating role of age in the relationship between ATS, religious coping mechanisms, and HRQoL has received limited attention, particularly in LMIC contexts.

**Figure 1:**
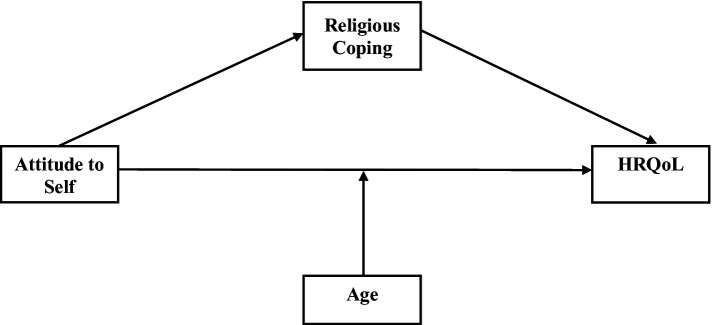
Conceptual frameworks.

This study aims to address these gaps by exploring the relationships between ATS and HRQoL in cancer patients, with a focus on the mediating role of religious coping and the moderating role of age. We therefore propose that attitude to self, which encompasses negative generalisation, self-criticism, and high standards, directly influences HRQoL. Religious coping mediates this relationship, with positive religious coping expected to improve HRQoL and negative religious Coping to impair it. In addition, we hypothesise that age could moderate these effects, with older individuals more vulnerable to the negative impacts of Attitude to Self and Negative Religious Coping and less likely to benefit from positive religious coping or High Standards. This framework highlights the interplay of cognitive vulnerabilities, coping strategies, and demographic factors (age) in shaping HRQoL among cancer patients (Figure 1). By examining these relationships, this study seeks to contribute to the understanding of cognitive vulnerabilities and coping strategies in cancer patients, particularly in LMICs, where culturally sensitive and age-specific interventions are critically needed.

## Materials and methods

### Procedures and participants

The Health Research Ethics Committee of the University Teaching Hospital in Southwest Nigeria University approved the study protocol. Between January 2019 and July 2019, cancer patients (*N* = 565) receiving oncology treatment at the teaching hospital were recruited. The January to July 2019 timeframe was chosen to ensure a sufficiently large sample size of 565 cancer patients, considering the patient volume and availability during that period at the University Teaching Hospital. While this period might limit capturing potential seasonal variations in patient experiences, the hospital’s continuous patient intake throughout the year suggests that the sample reasonably represents the cancer population receiving treatment at this facility.

Participants were recruited using purposive sampling, with individuals intentionally selected based on specific eligibility criteria, such as a cancer diagnosis. Eligibility criteria required participants to have a cancer diagnosis of any type or stage, be aged 18 years or older, and be able to read and understand English. Participants were excluded if they had severe psychiatric conditions or experienced extreme fatigue that could impair their ability to complete the questionnaires.

On clinic days, research assistants informed patients about the study’s purpose and procedures while they waited to see their physicians. The study was explained, and patients provided written informed consent to participate in the study. Six hundred questionnaires were distributed, each requiring approximately 15 min to complete. Completed questionnaires were collected immediately after completion. Of the 600 questionnaires distributed, 585 were returned (97.5% response rate). Twenty incomplete questionnaires were excluded, with 565 valid responses for data analysis. The sample included 189 males (33.5%) and 376 females (66.5%), with a mean age of 56.61 years (SD = 10.17). Most participants were married (88.5%). Additional demographic and clinical characteristics are summarised in Table 1.

**Table 1:** Demographic characteristics of participants.

Variable	Category	Frequency	Percent	Mean	SD
Age		18–77 (range)		55.61	10.17
Gender	Male	189	33.5		
	Female	376	66.5		
Marital Status	Married	500	88.5		
	Single	65	11.5		
Educational Level	No formal education	26	4.6		
	Primary education	67	11.9		
	Secondary education	234	41.4		
	Tertiary education	238	42.1		
Cancer Diagnosis	Breast cancer	183	32.4		
	Gynecological cancer	112	19.8		
	Prostrate cancer	78	13.8		
	Colon cancer	52	9.2		
	Lung cancer	35	6.2		
	Rectal cancer	22	3.9		
	Liver cancer	13	2.3		
	Squamous cell carcinoma	10	1.8		
	Gastric cancer	11	1.9		
	Others	49	8.9		
Stage of Cancer	Stage 1	70	12.4		
	Stage 2	257	45.5		
	Stage 3	224	39.6		
	Stage 4	14	2.5		
Time since Diagnosis	Less than 1 year	67	11.9		
	1 to 5 years	425	75.2		
	6 to 10 years	72	12.7		
	Over 10 years	1	0.2		
Current treatment	Chemotherapy				
	Yes	335	59.3		
	No	230	40.7		
	Radiotherapy				
	Yes	41	7.7		
	No	524	92.7		
	Surgery				
	Yes	380	67.3		
	No	185	32.7		

### Measures

#### The attitudes toward self-revised

The ATS-R (Carver et al., 1988) is a 10-item scale designed to assess three cognitive vulnerabilities associated with depression: High Standards (setting excessively high expectations, e.g., “Compared to other people, I expect a lot from myself”), Self-Criticism (tendency to criticize oneself for not meeting expectations, e.g., “When I do not do as well as I hoped to, I often get upset with myself.”), and Negative Generalization (generalizing a single failure to one’s overall self-worth, e.g., “If I notice one fault of mine, it makes me think about my other faults”). This revised version expands on the earlier scale (Carver et al., 1985) by including items that more explicitly target cognitive tendencies (Eisner et al., 2008). Participants rate items using a 5-point Likert scale ranging from 1 (I agree a lot) to 5(I disagree a lot), with higher scores indicating greater cognitive vulnerability in each domain. In this study, the scores for each subscale were summed and utilized. Cronbach’s alpha values for this study were 0.88 for High Standards, 0.76 for Self-Criticism, and 0.67 for Generalization.

#### Functional assessment of cancer therapy-general

The FACT-G (Cella et al., 1993) is a 27-item scale widely used to assess HRQoL. It consists of four subscales: physical well-being (7 items), social/family well-being (7 items), emotional well-being (6 items), and functional well-being (7 items). Each item is rated on a 5-point Likert scale, ranging from 0 (Not at all) to 4 (Very much). Subscale scores are calculated by summing the respective item scores, while the overall FACT-G score is derived by summing the scores of all four subscales. The total score ranges from 0 to 108, with higher scores indicating better quality of life (Cella et al., 1993; Brady et al., 1997). In this study, the sub-scores of the FACT-G score were utilized. The reliability of the scale is well-established, with the developers reporting a Cronbach’s alpha of 0.89 for the total score and subscale reliabilities ranging from 0.69 to 0.82 (Cella et al., 1993). In this study, the total FACT-G Cronbach’s alpha was 0.91, with subscale reliabilities ranging from 0.78 to 0.89.

#### The brief religious coping scale

The Brief-COPE (Pargament et al., 2001) is a 14-item measure derived from the original Religious Coping Scale (RCOPE) to assess religious coping strategies. It consists of two subscales: positive religious coping (seven items) and Negative Religious Coping (seven items). Positive religious coping evaluates a secure and trusting relationship with God, while NRC reflects a religious struggle stemming from a more fragile connection with God (Pargament et al., 2004). Responses are recorded on a 4-point Likert scale ranging from 1 (a great deal) to 4 (not at all). Higher scores indicate greater use of either positive or negative religious coping. The Brief RCOPE has demonstrated strong reliability and validity in previous research (Pargament et al., 2011), including studies with cancer patients (Sherman et al., 2005). In this study, Cronbach’s alpha coefficients were 0.84 for positive religious coping and 0.88 for negative religious coping.

### Design and statistics

This study employed a cross-sectional design to investigate the relationships among cancer patients’ attitudes toward the self (ATS), religious coping, age, and health-related quality of life (HRQoL). The research specifically examined the mediating role of religious coping, which included positive and negative coping strategies, and the moderating effect of age in the relationship between ATS dimensions (High Standards, Negative Generalization, and Self-Criticism) and HRQoL outcomes.

The statistical procedures were conducted in four steps. First, descriptive statistics and correlation analyses were conducted to summarize the data and examine relationships among demographic characteristics, ATS dimensions, religious coping styles, and HRQoL domains. Means, standard deviations, and Pearson correlation coefficients were computed.

Second, hierarchical multiple regression analyses evaluated the direct effects of ATS dimensions, religious coping, and age on HRQoL domains. The regression models entered variables in the following order: (1) demographic variables (age, gender, marital status), (2) ATS dimensions, and (3) religious coping styles to predict HRQoL.

Third, the moderating effect of age was tested. Age was centered on minimizing multicollinearity, and interaction terms were created by multiplying age with ATS dimensions and religious coping styles. Simple slope analysis was conducted to interpret significant interactions and better understand how the relationships between variables differed across age groups.

Fourth, mediation analyses were performed to examine whether positive and negative religious coping mediated the relationships between ATS dimensions and HRQoL domains. Serial multiple mediation models were implemented using SmartPLS, with bootstrapping (10,000 resamples) employed to calculate bias-corrected 95% confidence intervals for the indirect effects. A mediation effect was considered significant if the confidence intervals did not include zero. The data analysis was conducted using SPSS (Version 29) and SmartPLS (Version 4). To ensure robust conclusions, all analyses controlled for gender, marital status, educational level, cancer diagnosis, stage of cancer, time since diagnosis, and current treatment. The models included these covariates to account for their potential confounding effects on well-being outcomes.

The correlation matrix (Table 2) revealed complex associations between demographic characteristics, attitude to self, religious coping styles, and multiple dimensions of well-being. Age was negatively correlated with physical well-being (PWB), *r* = −0.08, *p* < 0.05, suggesting that older participants reported slightly lower physical health. Gender differences emerged, with women reporting higher emotional well-being (EWB; *r* = 0.17, *p* < 0.01) and physical well-being (PWB; *r* = 0.12, *p* < 0.01), yet lower social/family well-being (SWB; *r* = −0.13, *p* < 0.01) compared to men.

**Table 2:** Descriptive and correlation matrix.

		M	SD	1	2	3	4	5	6	7	8	9	10	11	12	13
1	Age	56.61	10.17	--												
2	Gender			−0.05	--											
3	Marital status			−0.34^**^	−0.13^**^	--										
4	Educational level	2.21	0.83	−0.23^**^	−0.23^**^	0.05	--									
5	Positive religious coping	17.79	2.60	0.00	−0.07	−0.15^**^	0.06	--								
6	Negative religious coping	6.58	2.57	−0.09^*^	0.03	0.15^**^	−0.08	−0.47^**^	--							
7	High standard	9.02	3.83	−0.12^**^	0.06	0.04	−0.09^*^	−0.26^**^	0.33^**^	--						
8	Negative Generalization	11.23	2.35	−0.05	−0.06	0.16^**^	−0.03	−0.02	0.18^**^	0.14^**^	--					
9	Self-criticism	10.27	2.93	−0.02	−0.03	0.08	0.01	0.08	−0.02	0.01	0.47^**^	--				
10	Physical well-being	14.84	6.44	−0.08^*^	0.12^**^	−0.17^**^	0.06	0.11^**^	−0.24^**^	0.04	−0.31^**^	−0.28^**^	--			
11	Social/family wellbeing	21.32	5.31	0.07	−0.13^**^	−0.01	0.04	0.26^**^	−0.42^**^	−0.16^**^	−0.03	0.21^**^	0.06	--		
12	Emotional wellbeing	13.02	4.80	−0.02	0.17^**^	−0.23^**^	−0.01	−0.02	−0.10^*^	0.25^**^	−0.13^**^	−0.18^**^	0.52^**^	−0.11^**^	--	
13	Functional wellbeing	15.77	6.79	−0.08	0.09^*^	−0.10^*^	0.06	0.20^**^	−0.32^**^	−0.13^**^	−0.27^**^	−0.03	0.50^**^	0.53^**^	0.25^**^	--

**p < 0.01, *p < 0.05. Gender (dummy coded ‘0’-Male, ‘1’-Female); MS (Marital status), (dummy coded ‘0’-Single, ‘1’-Married); EL, Educational level; SC, Self-criticism; NG, Negative Generalization; HS, High standard; NRC, Negative religious coping; PRC, Positive religious coping; PWB, Physical well-being; SWB, Social/family wellbeing; EWB, Emotional wellbeing; FWB, Functional wellbeing.

Marital status demonstrated a negative correlation with PWB (*r* = −0.17, *p* < 0.01), EWB (*r* = −0.23, *p* < 0.01), and functional well-being (FWB; *r* = −0.10, *p* < 0.05), indicating that married individuals tended to experience slightly lower well-being in these domains. Positive religious coping was consistently linked to beneficial outcomes, including higher PWB (*r* = 0.11, *p* < 0.01), SWB (*r* = 0.26, *p* < 0.01), and FWB (*r* = 0.20, *p* < 0.01). In contrast, negative religious coping showed negative associations with several well-being dimensions, including PWB (*r* = −0.24, *p* < 0.01), SWB (*r* = −0.42, *p* < 0.01), and FWB (*r* = −0.32, *p* < 0.01), suggesting that this maladaptive religious coping style undermines multiple facets of health.

The cognitive variables (attitude to self) of high standards, negative generalization, and self-criticism also displayed notable patterns. HS was linked to higher EWB (*r* = 0.25, *p* < 0.01) but lower SWB (*r* = −0.16, *p* < 0.01) and FWB (*r* = −0.13, *p* < 0.01). Negative generalization was negatively correlated with PWB (*r* = −0.31, *p* < 0.01), EWB (*r* = −0.13, *p* < 0.01), and FWB (*r* = −0.27, *p* < 0.01), indicating that a tendency to generalize negative experiences relates to poorer well-being across multiple domains. Self-Criticism was negatively associated with PWB (*r* = −0.28, *p* < 0.01) and EWB (*r* = −0.18, *p* < 0.01) but positively correlated with SWB (*r* = 0.21, *p* < 0.01). This finding may suggest that while self-criticism can be detrimental to emotional and physical health, it may simultaneously be linked with enhanced relational or familial support.

Table 3 presents the direct effects of age, negative generalization, high standards, self-criticism, and religious coping (positive and negative) on various well-being outcomes and the moderation of these effects by age. Negative generalization significantly reduced physical well-being (PWB, *b* = −0.19, *p* < 0.001) and functional well-being (FWB, *b* = −0.25, *p* < 0.001), accounting for 19 and 25% of the variance in these domains, respectively. These findings highlight the detrimental effects of negative cognitive patterns, suggesting that interventions targeting maladaptive thoughts could significantly improve physical and functional health. Negative generalization also increased reliance on negative religious coping (NRC, *b* = 0.17, *p* < 0.01), further compounding its adverse effects on health-related quality of life (HRQoL).

**Table 3:** Direct effect of attitude to self, religious coping, age and well-being.

Direct effect	*b*	*t*	*p*	95% CI (LL, UL)
Negative Generalization → Emotional Well-Being	−0.06	1.25	0.11	[−0.144, 0.025]
Negative Generalization → Functional Well-Being	−0.25	4.71	0.01	[−0.336, −0.161]
Negative Generalization → Physical Well-Being	−0.19	4.37	0.01	[−0.255, −0.114]
Negative Generalization → Social/family wellbeing	−0.08	1.68	0.04	[−0.157, −0.004]
Negative Generalization → Negative religious coping	0.17	3.48	0.01	[0.089, 0.253]
Negative Generalization → Positive religious coping	−0.03	0.63	0.26	[−0.123, 0.052]
High standard → Emotional Well-Being	0.27	7.24	0.01	[0.212, 0.335]
High standard → Functional Well-Being	0.00	0.01	0.50	[−0.061, 0.051]
High standard → Physical Well-Being	0.16	4.82	0.01	[0.100, 0.206]
High standard → Social/family wellbeing	0.04	1.22	0.11	[−0.013, 0.084]
High standard → Negative religious coping	0.20	5.84	0.01	[0.143, 0.255]
High standard → Positive religious coping	−0.26	7.15	0.01	[−0.316, −0.197]
Self-criticism → Emotional Well-Being	−0.16	3.51	0.01	[−0.231, −0.084]
Self-criticism → Functional Well-Being	0.10	2.11	0.02	[0.021, 0.168]
Self-criticism → Physical Well-Being	−0.19	4.33	0.01	[−0.257, −0.116]
Self-criticism → Social/family wellbeing	0.24	5.63	0.01	[0.167, 0.305]
Self-criticism → Negative religious coping	−0.07	1.78	0.04	[−0.125, −0.005]
Self-criticism → Positive religious coping	0.10	1.93	0.03	[0.014, 0.182]
Negative religious coping → Emotional Well-Being	−0.20	4.05	0.01	[−0.275, −0.114]
Negative religious coping → Functional Well-Being	−0.25	5.10	0.01	[−0.329, −0.165]
Negative religious coping → Physical Well-Being	−0.25	5.35	0.01	[−0.326, −0.172]
Negative religious coping → Social/family wellbeing	−0.37	7.11	0.01	[−0.455, −0.284]
Positive religious coping → Emotional Well-Being	−0.050	1.07	0.14	[−0.116, 0.035]
Positive religious coping → Functional Well-Being	0.09	1.59	0.06	[0.000, 0.182]
Positive religious coping → Physical Well-Being	0.07	1.48	0.07	[−0.008, 0.143]
Positive religious coping → Social/family wellbeing	0.11	2.04	0.02	[0.017, 0.190]
Positive religious coping → Negative religious coping	−0.41	9.78	0.01	[−0.475, −0.339]
Age → Emotional Well-Being	−0.03	0.68	0.25	[−0.092, 0.040]
Age → Functional Well-Being	−0.11	2.48	0.01	[−0.173, −0.031]
Age → Physical Well-Being	−0.09	2.23	0.01	[−0.153, −0.017]
Age → Social/family wellbeing	0.06	1.51	0.07	[−0.004, 0.132]
Age × Negative Generalization → Emotional Well-Being	−0.07	1.38	0.08	[−0.157, 0.009]
Age × Negative Generalization → Functional Well-Being	−0.06	1.02	0.15	[−0.154, 0.025]
Age × Negative Generalization → Physical Well-Being	−0.09	2.17	0.02	[−0.161, −0.023]
Age × Negative Generalization → Social/family wellbeing	−0.04	0.92	0.18	[−0.129, 0.030]
Age × Self-criticism → Emotional Well-Being	0.07	1.30	0.10	[−0.02, 0.142]
Age × Self-criticism → Functional Well-Being	−0.09	1.93	0.03	[−0.168, −0.012]
Age × Self-criticism → Physical Well-Being	−0.10	2.46	0.01	[−0.173, −0.034]
Age × Self-criticism → Social/family wellbeing	−0.04	0.78	0.22	[−0.105, 0.042]
Age × High standard → Emotional Well-Being	0.03	0.61	0.27	[−0.043, 0.094]
Age × High standard → Functional Well-Being	−0.10	2.47	0.01	[−0.157, −0.030]
Age × High standard → Physical Well-Being	−0.12	3.07	0.01	[−0.178, −0.053]
Age × High standard → Social/family wellbeing	−0.06	1.87	0.03	[−0.122, −0.010]

*b, standardized indirect effect; SE, standard error; t, test statistic; p, p-value; CI, confidence interval; LL, lower limit; UL, upper limit of the 95% CI.*

High standards demonstrated a dual effect. While they positively influenced emotional well-being (EWB, *b* = 0.27, *p* < 0.001) and physical well-being (*b* = 0.16, *p* < 0.001), explaining 27 and 16% of the variance, respectively, they negatively impacted FWB (*b* = −0.13, *p* < 0.01), with 13% variance explained. Moreover, high standards were positively associated with NRC (*b* = 0.20, *p* < 0.001) and negatively with positive religious coping (PRC, *b* = −0.26, *p* < 0.001). These results suggest that while high standards promote resilience, their maladaptive implementation may promote spiritual struggles and reduce emotional and functional well-being.

Self-criticism negatively predicted PWB (*b* = −0.19, *p* < 0.001) and EWB (*b* = −0.16, *p* < 0.001), accounting for 19 and 16% of the variance in these outcomes. Interestingly, self-criticism positively influenced FWB (*b* = 0.10, *p* = 0.02) and social/family well-being (SWB, *b* = 0.24, *p* < 0.001), explaining 24% of the variance in SWB. These findings indicate that in collectivist cultures, self-critical tendencies may drive social engagement and support, partially offsetting their negative effects on emotional and physical well-being.

Religious coping styles also played a critical role. NRC had pervasive negative effects on all HRQoL domains. Specifically, NRC significantly reduced EWB (*b* = −0.20, *p* < 0.01), FWB (*b* = −0.25, *p* < 0.001), PWB (*b* = −0.25, *p* < 0.001), and SWB (*b* = −0.37, *p* < 0.001), with SWB experiencing the most pronounced impact, accounting for 37% of the variance. These findings emphasize NRC’s role in undermining psychological, functional, and relational health, suggesting that spiritual struggles exacerbate stress responses and reduce overall well-being.

Conversely, PRC demonstrated a more targeted influence, significantly enhancing SWB (*b* = 0.11, *p* = 0.02) and explaining 11% of the variance. However, its effects on other HRQoL domains, such as EWB, FWB, and PWB, were not statistically significant. This pattern indicates that PRC may primarily strengthen social and relational well-being without sufficiently counteracting broader negative impacts associated with cognitive vulnerabilities.

Age demonstrated significant negative direct effects on functional well-being (FWB), *b* = −0.11, *t* = 2.48, *p* = 0.01, 95% CI [−0.173, −0.031], and physical well-being (PWB), *b* = −0.09, *t* = 2.23, *p* = 0.01, 95% CI [−0.153, −0.017]. Although age did not have a significant direct effect on emotional well-being (EWB) or social/family well-being (SWB), it did interact significantly with NG to predict PWB, *b* = −0.09, *t* = 2.17, *p* = 0.02, 95% CI [−0.161, −0.023], and with High standard to predict FWB, PWB, and SWB, suggesting that the negative impact of these cognitive styles on well-being became more pronounced with increasing age.

The moderation analyses examined how age influenced the relationships between cognitive predictors, Negative Generalization, High Standards, and Self-Criticism, and various well-being outcomes, including Emotional Well-Being (EWB), Functional Well-Being (FWB), Physical Well-Being (PWB), and Social/Family Well-Being (SWB).

**Figure 2:**
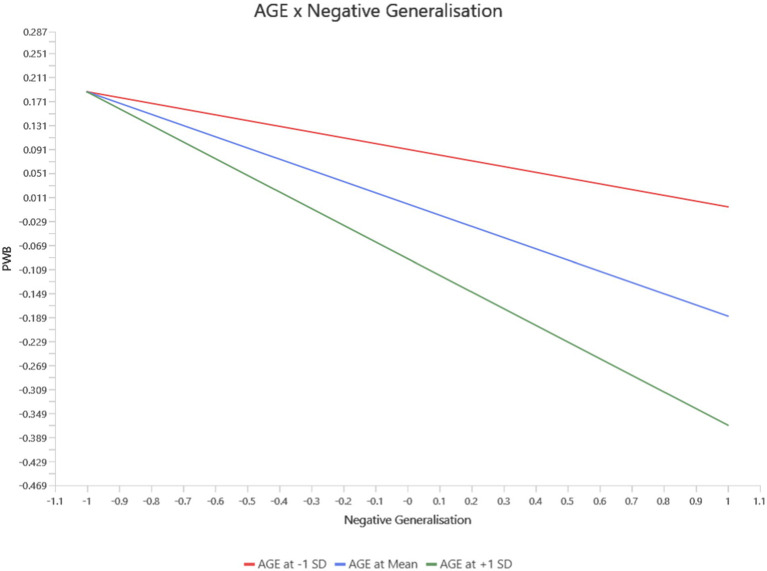
Interaction between age and negative generalization on PWB.

For Negative Generalization, a significant interaction with age was observed for Physical Well-Being (PWB). Older individuals with higher levels of negative generalization experienced a more pronounced decline in PWB than younger individuals (*b* = −0.09, *p* = 0.02, 95% CI [−0.161, −0.023]). This finding suggests that the detrimental effects of negative cognitive patterns on physical health become more severe with age (see Figure 2).

**Figure 3:**
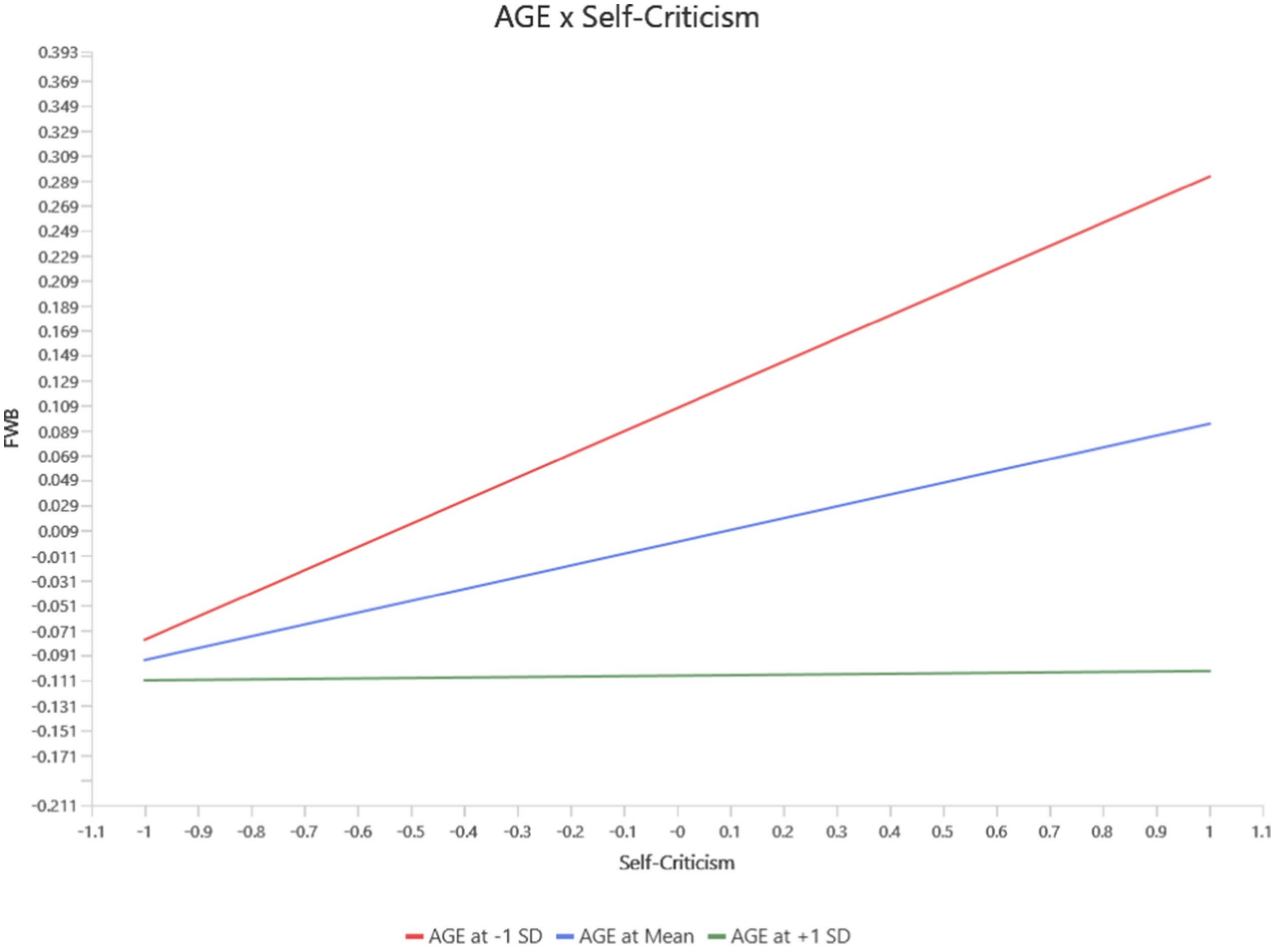
Interaction between age and self-criticism on FWB.

**Figure 4:**
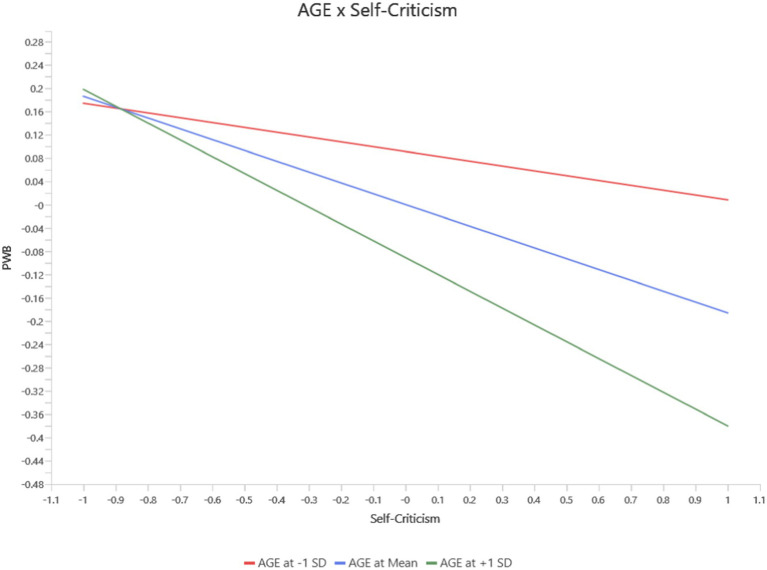
Interaction between age and self-criticism on PWB.

Self-criticism showed significant age-moderated effects on Functional Well-Being (FWB) and PWB. At higher age levels, Self-criticism had a stronger negative impact on FWB (*b* = −0.09, *p* = 0.03, 95% CI [−0.168, −0.012]) (see Figure 3) and PWB (*b* = −0.10, *p* = 0.01, 95% CI [−0.173, −0.034]) (see Figure 4). These findings indicate that self-critical tendencies disproportionately affect functional and physical health among older individuals, while these relationships were weaker or non-significant at lower age levels.

**Figure 5:**
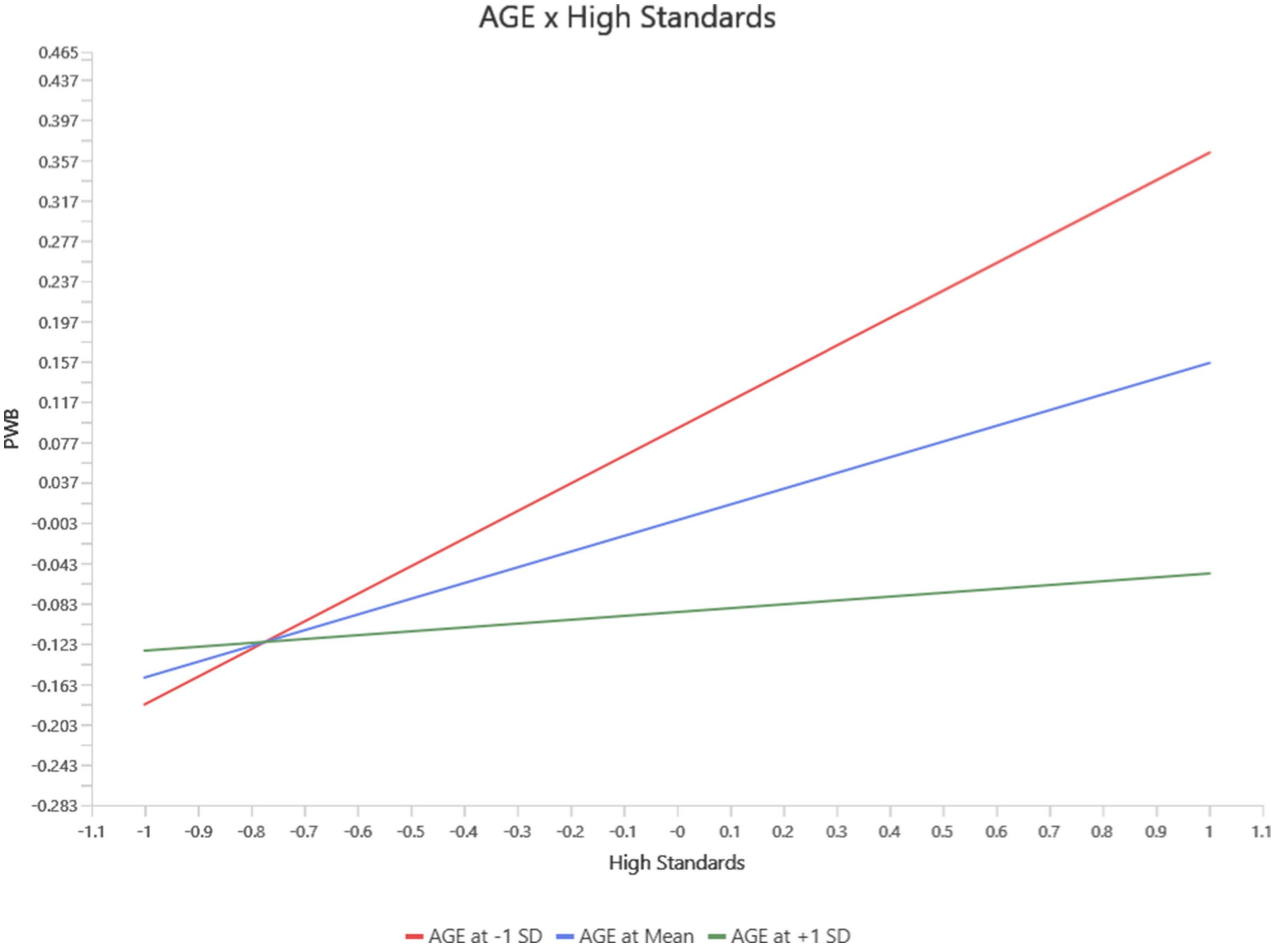
Interaction between age and high standard on PWB.

**Figure 6:**
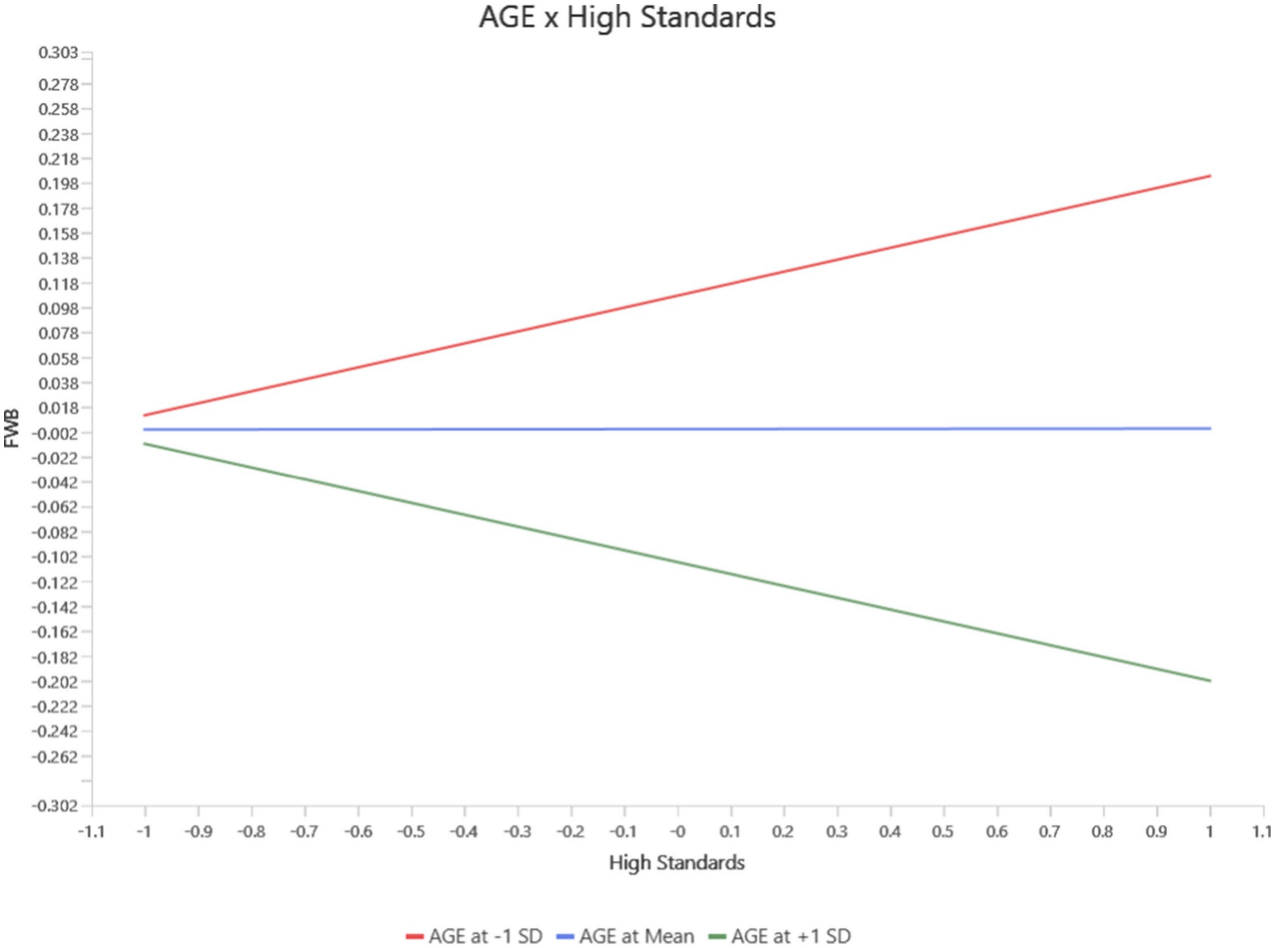
Interaction between age and high standard on FWB.

**Figure 7:**
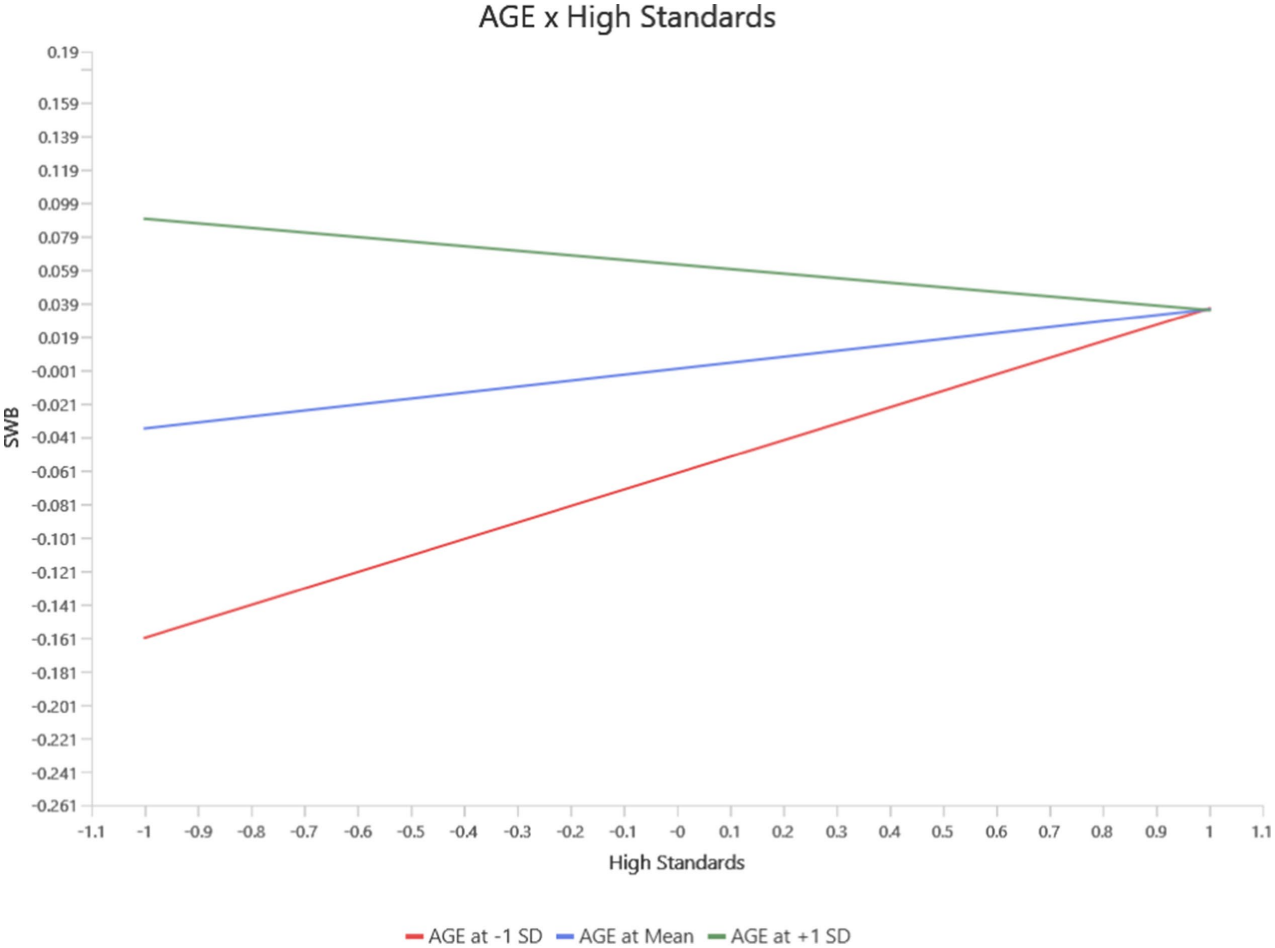
Interaction between age and high standard on SWB.

For High Standards, age moderated its effects on Functional Well-Being (FWB), PWB, and Social/Family Well-Being (SWB). At younger ages, High standards positively predicted PWB (*b* = 0.23, *p* = 0.03, 95% CI [0.02, 0.44]). However, this protective effect diminished with increasing age and became non-significant at mean age levels and slightly negative at high age levels (see Figure 5). Similarly, the negative impact of high standard on FWB became significant at the mean and high age levels (*b* = −0.10, *p* = 0.01, 95% CI [−0.157, −0.030]), while no significant effects were observed at younger ages levels (see Figure 6). For SWB, high standards had a negligible effect at younger ages but exhibited a significant negative relationship at the mean and high age levels (*b* = −0.06, *p* = 0.03, 95% CI [−0.122, −0.010]) levels (see Figure 7).

Overall, the findings highlight that age amplifies the negative effects of High Standards, Self-Criticism, and negative generalization on well-being outcomes, particularly for FWB and PWB. Older individuals are more vulnerable to the harmful impacts of these cognitive predictors, while younger individuals may experience some protective effects, particularly from high standards. This underlines the importance of considering age as a moderating factor when addressing the interplay between cognitive styles and well-being.

A series of serial multiple mediation analyses were conducted to explore whether positive religious coping and negative religious coping mediated the relationships between cognitive predictors (Self-Criticism, Negative Generalization, and High Standards) and various well-being outcomes, including Emotional Well-Being (EWB), Functional Well-Being (FWB), Physical Well-Being (PWB), and Social/Family Well-Being (SWB) (see Table 4). The results revealed significant and distinct patterns for each predictor.

**Table 4:** Specific indirect effects of predictors on well-being outcomes via PRC and NRC.

Predictor → Mediator(s) → Outcome	*b*	*t*	*p*	95% CI (LL, UL)
Negative Generalization → PRC → EWB	0.00	0.46	0.32	[0.00, 0.01]
Negative Generalization → PRC → FWB	0.00	0.52	0.30	[−0.01, 0.01]
Negative Generalization → PRC → PWB	0.00	0.47	0.32	[−0.01, 0.00]
Negative Generalization → PRC → SWB	0.00	0.58	0.28	[−0.01, 0.01]
Negative Generalization → NRC → EWB	−0.04	2.97	0.00	[−0.05, −0.02]
Negative Generalization → NRC → FWB	−0.04	2.65	0.00	[−0.07, −0.02]
Negative Generalization → NRC → PWB	−0.04	3.30	0.00	[−0.07, −0.02]
Negative Generalization → NRC → SWB	−0.06	2.88	0.00	[−0.10, −0.03]
High Standards → PRC → NRC → EWB	−0.02	3.07	0.00	[−0.03, −0.01]
High Standards → PRC → NRC → FWB	−0.03	4.14	0.00	[−0.04, −0.02]
High Standards → PRC → NRC → PWB	−0.03	3.61	0.00	[−0.04, −0.02]
High Standards → PRC → NRC → SWB	−0.04	5.01	0.00	[−0.05, −0.03]
Self-Criticism → PRC → NRC → EWB	0.01	1.72	0.04	[0.01, 0.02]
Self-Criticism → PRC → NRC → FWB	0.01	1.78	0.04	[0.01, 0.02]
Self-Criticism → PRC → NRC → SWB	0.02	1.89	0.03	[0.01, 0.03]
Self-Criticism → PRC → NRC → PWB	0.01	1.79	0.04	[0.01, 0.02]
High Standards → PRC → EWB	0.01	1.06	0.14	[−0.01, 0.03]
High Standards → PRC → FWB	−0.02	1.51	0.07	[−0.05, 0.00]
High Standards → PRC → PWB	−0.02	1.45	0.07	[−0.04, 0.00]
High Standards → PRC → SWB	−0.03	1.90	0.03	[−0.05, 0.00]
High Standards → NRC → EWB	−0.04	3.39	0.00	[−0.06, −0.02]
High Standards → NRC → FWB	−0.05	4.02	0.00	[−0.07, −0.03]
High Standards → NRC → PWB	−0.05	4.22	0.00	[−0.07, −0.03]
High Standards → NRC → SWB	−0.07	4.61	0.00	[−0.10, −0.05]
Self-Criticism → PRC → PWB	0.01	1.04	0.15	[0.00, 0.02]
Self-Criticism → PRC → SWB	0.01	1.36	0.09	[0.00, 0.03]
Self-Criticism → PRC → EWB	−0.01	0.87	0.19	[−0.01, 0.00]
Self-Criticism → PRC → FWB	0.01	1.22	0.11	[0.00, 0.02]
Self-Criticism → NRC → EWB	0.01	1.58	0.06	[0.00, 0.03]
Self-Criticism → NRC → FWB	0.02	1.72	0.04	[0.01, 0.03]
Self-Criticism → NRC → PWB	0.02	1.62	0.05	[0.00, 0.03]
Self-Criticism → NRC → SWB	0.02	1.79	0.04	[0.01, 0.05]
Negative Generalization → PRC → NRC → EWB	0.00	0.63	0.27	[−0.01, 0.00]
Negative Generalization → PRC → NRC → FWB	0.00	0.61	0.27	[−0.01, 0.01]
Negative Generalization → PRC → NRC → PWB	0.00	0.63	0.27	[−0.01, 0.01]
Negative Generalization → PRC → NRC → SWB	−0.01	0.61	0.27	[−0.02, 0.01]

*b, unstandardized indirect effect; CI, confidence interval; LL, lower limit; UL, upper limit; PRC, Positive Religious Coping; NRC, Negative Religious Coping; EWB, Emotional Well-Being; FWB, Functional Well-Being; PWB, physical Well-Being; SWB, Social/Family Well-Being.*

Negative Generalization did not show significant serial mediation effects through positive religious coping and negative religious coping. However, it had notable indirect effects on all well-being outcomes via negative religious coping alone. A stronger tendency to generalize negatively was associated with increased use of negative religious coping, which negatively impacted EWB (*b* = −0.04, *p* < 0.001, 95% CI [−0.05, 0.02]), FWB (*b* = −0.04, *p* < 0.001, 95% CI [−0.07, −0.02]), PWB (*b* = −0.04, *p* < 0.001, 95% CI [−0.07, −0.02]), and SWB (*b* = −0.06, *p* < 0.001, 95% CI [−0.10, −0.03]). This highlights the detrimental role of negative coping patterns in exacerbating poor well-being outcomes.

High Standards, in contrast, had negative indirect effects on well-being outcomes via positive religious coping and negative religious coping. Individuals with high standards were less likely to engage in positive religious coping and more likely to rely on negative religious coping, which led to decreased EWB (*b* = −0.02, *p* < 0.001, 95% CI [−0.03, −0.01]), FWB (*b* = −0.03, *p* < 0.001, 95% CI [−0.04, −0.02]), PWB (*b* = −0.03, *p* < 0.001, 95% CI [−0.04, −0.02]), and SWB (*b* = −0.04, *p* < 0.001, 95% CI [−0.05, −0.03]). This indicates that holding oneself to high standards may undermine well-being by promoting maladaptive coping patterns.

Self-criticism demonstrated significant indirect effects on all well-being outcomes through positive and negative religious coping. Specifically, higher levels of self-criticism were associated with increased positive religious coping and decreased negative religious coping, which in turn improved EWB (*b* = 0.01, *p* = 0.04, 95% CI [0.01, 0.02]), FWB (*b* = 0.01, *p* = 0.04, 95% CI [0.01, 0.02]), SWB (*b* = 0.02, *p* = 0.03, 95% CI [0.01, 0.03]), and PWB (*b* = 0.01, *p* = 0.04, 95% CI [0.01, 0.02]). These findings suggest that self-critical individuals may experience enhanced well-being indirectly by engaging in more adaptive religious coping strategies (positive religious coping) while minimizing the use of maladaptive coping strategies (negative religious coping).

## Discussion

This study explored the relationships between attitudes toward the self (ATS), health-related quality of life (HRQoL), the mediating effect of religious coping, and the moderating role of age among cancer patients. Our findings underscore the complex interactions between cognitive vulnerabilities, religious coping mechanisms, and age in shaping HRQoL, particularly in low- and middle-income contexts. The results highlight the significant role of ATS dimensions in shaping HRQoL, extending prior research by demonstrating the complex effects of these dimensions in a cancer population. Negative Generalisation showed strong adverse effects on physical well-being (PWB), functional well-being (FWB), and social/family well-being (SWB), consistent with cognitive vulnerability theories (Abramson et al., 2002). However, negative generalisation’s non-significant effect on emotional well-being (EWB) suggests that factors such as coping styles or external support may moderate its influence in this domain. High Standards exhibited a dual role, positively predicting EWB and PWB. While maintaining high expectations can promote resilience and motivation, these benefits may diminish when tied to punitive coping strategies (Carver et al., 1985). Conversely, Self-Criticism negatively influenced PWB and EWB but positively predicted SWB and FWB. This dual effect may reflect cultural dynamics in collectivist settings, where self-critical tendencies elicit greater social support and communal engagement. To the best of our knowledge, this is the first study to examine the impact of attitudes toward the self (ATS) on health-related quality of life (HRQoL) in cancer patients, highlighting the critical role of cognitive vulnerability in this population.

The mediating role of religious coping was evident, particularly for negative religious coping. The HRQoL dimensions referenced here include physical well-being (PWB), functional well-being (FWB), emotional well-being (EWB), and social/family well-being (SWB). Negative religious coping significantly mediated the relationships between Negative generalisation and Self-criticism with these HRQoL dimensions, reinforcing its detrimental impact. This finding aligns with studies demonstrating that negative religious coping mediates the relationship between perceived stress and health-related quality of life (HRQoL) among cancer patients (Okwuosa et al., 2024). Beyond its mediating role, Negative religious coping exhibited a negative relationship with HRQoL, supporting evidence that it exacerbates psychological distress by amplifying stress responses, reducing adaptive resources, and disrupting emotional functioning (Pargament et al., 2011). Specifically, negative religious coping was found to undermine physical well-being (PWB) and functional well-being significantly (FWB), with its pervasive negative effects extending to social well-being (SWB), further highlighting its detrimental impact across these domains. By contrast, positive religious coping’s mediating role was limited, with notable effects only observed for high standards on SWB. The weaker influence of PRC on these dimensions may stem from contextual factors or patient characteristics. For instance, the PRC might require specific supportive environments, such as strong community or familial networks, to translate into tangible benefits for broader aspects of HRQoL. Additionally, the impact of PRC could vary depending on patient characteristics like age or cancer stage. Older individuals or those at more advanced cancer stages might prioritise spiritual and relational well-being over physical or emotional domains, thereby limiting the observable effects of PRC on these latter dimensions. Alternatively, the perceived benefits of PRC might be more gradual and cumulative, which could be more evident in longitudinal studies. Future research should explore these contextual and individual differences to better understand the conditions under which the PRC influences HRQoL.

Age emerged as a significant moderator, amplifying the adverse effects of negative generalisation and self-criticism on HRQoL. This highlights the importance of age-sensitive interventions, such as tailored community support programs and therapies addressing the unique vulnerabilities associated with ageing. This finding aligns with socioemotional selectivity theory, which posits that older adults prioritise immediate emotional well-being but may experience heightened vulnerability to stressors and physical decline (Carstensen, 2021). The adaptive benefits of high standards on PWB, evident at younger ages, diminished with age, ultimately turning negative. This indicates that high standards may become counterproductive for older individuals grappling with significant health and functional challenges. The indirect effects of negative religious coping were more pronounced in older participants, underscoring age-related vulnerabilities in managing maladaptive coping strategies. While positive religious coping showed limited age-related variation, the findings highlight the pressing need to address negative religious coping in older cancer patients to mitigate its adverse effects and improve HRQoL.

In summary, the relationships between negative generalization, self-criticism, and HRQoL are mediated by religious coping styles and moderated by age. These findings emphasize the importance of culturally and contextually tailored interventions to improve HRQoL among cancer patients. Targeted strategies addressing both cognitive vulnerabilities (e.g., high standards and self-criticism) and maladaptive coping mechanisms (e.g., NRC) could significantly enhance resilience and well-being in this population. Future research should consider longitudinal designs to explore the temporal dynamics of these relationships and evaluate the efficacy of intervention programs in diverse cultural settings.

## Theoretical and practical implications of the findings

This study advances understanding of cognitive vulnerabilities, religious coping, and HRQoL among cancer patients, particularly in low- and middle-income contexts. The findings underscore the importance of cognitive-behavioural interventions to reduce cognitive vulnerability in cancer patients and survivors. Techniques such as cognitive restructuring and self-compassion training can mitigate negative effects, while psychoeducational programmes should prioritise reducing negative religious coping and promoting adaptive religious coping strategies.

Clinicians must be equipped to identify and effectively address negative religious coping in therapeutic settings. Tailored interventions for older cancer patients must address their unique vulnerabilities, including physical decline and intensified stress responses. Additionally, culturally sensitive programmes integrating collectivist values and spiritual practices, such as community-based support systems, can enhance intervention efficacy. Prior studies have demonstrated the efficacy of culturally aligned coping strategies in enhancing psychological adjustment among cancer patients (Puchalska-Wasyl and Małaj, 2024).

## Strengths and limitations

This study offers robust contributions to understanding HRQoL among cancer patients. The large sample size and high response rate (97.5%) enhance the reliability and generalisability of the findings. Comprehensive assessments of ATS, religious coping, and HRQoL provide valuable insights into their interrelationships while including age as a moderator, highlighting a novel dimension in understanding HRQoL dynamics.

While the cross-sectional design of this study allowed for a snapshot of relationships between variables, it limits the ability to infer causation. For instance, the observed associations between coping strategies (NRC and PRC) and HRQoL outcomes could reflect bidirectional or cyclical relationships. A longitudinal approach would enable the exploration of causal pathways, such as whether NRC exacerbates declines in HRQoL over time or if worsening HRQoL leads to greater reliance on NRC. Additionally, a longitudinal design could reveal how the benefits of PRC unfold across different stages of cancer treatment and survivorship, offering insights into when and how interventions should be applied for maximum impact. Future longitudinal studies could address these gaps, providing a more nuanced understanding of the dynamic interplay between coping mechanisms and HRQoL.

Another limitation lies in using self-reported measures, which are inherently susceptible to response biases. For instance, social desirability bias may have influenced participants to underreport negative coping behaviors such as NRC or to overreport positive attributes like PRC and high standards. This could result in underestimating the negative impacts of maladaptive coping strategies or overestimating the protective effects of adaptive ones. Additionally, self-report measures rely on participants’ ability to accurately recall and evaluate their behaviors and experiences, which may introduce recall bias. Future studies could mitigate these limitations by incorporating objective measures, such as clinician ratings or biomarker data, to complement self-reported outcomes.

## Future research

Future research should adopt longitudinal designs to explore causal pathways, focusing on the dynamic interplay between ATS, coping mechanisms, and HRQoL over time. A mixed-methods approach, combining quantitative data with qualitative insights from interviews or focus groups, could enrich the understanding of cultural complexities in coping. Investigating PRC’s role across diverse cultural and clinical contexts will clarify its impact on well-being.

Research should also prioritize intervention studies to mitigate the adverse effects of maladaptive attitudes and coping mechanisms, such as NRC and excessive self-criticism. For example, spiritually integrated cognitive-behavioural therapy (CBT) could be tailored to help individuals reframe maladaptive religious beliefs and develop more adaptive spiritual practices. Interventions like mindfulness-based stress reduction (MBSR) and self-compassion training may also prove effective in reducing the emotional toll of high standards and self-criticism, particularly among patients experiencing heightened distress.

Group-based interventions focusing on peer support and community engagement could amplify the effects of PRC by fostering stronger social networks, which may enhance relational and emotional well-being for older adults or patients in advanced cancer stages, age- and stage-specific programs that address unique stressors and prioritise quality of life should be explored. Such interventions might include life review therapy or dignity therapy, which can help patients integrate their spiritual and emotional experiences into their coping strategies.

Future studies should examine additional moderators, including gender and cancer stage, as well as mediators, such as social support and optimism. Gender, in particular, may influence coping and HRQoL due to societal expectations and differing socialization patterns. For instance, women may be more likely to engage in emotionally expressive coping or rely on relational support, while men might prioritize problem-focused or avoidant coping strategies. These variations could shape interventions’ effectiveness and coping mechanisms’ impact on HRQoL. Exploring how gender interacts with coping strategies and ATS dimensions will provide a more nuanced understanding of these dynamics and inform the development of gender-sensitive interventions. Furthermore, developing and testing interventions tailored to ATS dimensions, coping strategies, and age-specific needs will enhance efforts to improve HRQoL in cancer patients.

## Conclusion

This study underlines the pivotal role of cognitive vulnerabilities, religious coping, and age in influencing HRQoL among cancer patients. The findings emphasise the need for targeted interventions that address maladaptive attitudes and coping strategies, particularly in older individuals, to enhance overall well-being. Moreover, the study highlights the significance of integrating age-sensitive and culturally relevant approaches, including adaptive religious coping, to improve the quality of life for cancer patients in low- and middle-income countries.

## Data Availability

The raw data supporting the conclusions of this article will be made available by the authors, without undue reservation.
